# Association of LncRNA‐GAS5 gene polymorphisms and PBMC LncRNA‐GAS5 level with risk of systemic lupus erythematosus in Chinese population

**DOI:** 10.1111/jcmm.16438

**Published:** 2021-03-16

**Authors:** Chun‐Hong Liu, Yu‐Lan Lu, Hua‐Tuo Huang, Chun‐Fang Wang, Hong‐Cheng Luo, Gui‐Jiang Wei, Ming Lei, Tan Tan, Yan Wang, Yan‐Yun Huang, Ye‐Sheng Wei, Yan Lan

**Affiliations:** ^1^ Department of Laboratory Medicine The Affiliated Hospital of Youjiang Medical University for Nationalities Baise China; ^2^ Department of Medical Reproduction Center The Affiliated Hospital of Youjiang Medical University for Nationalities Baise China; ^3^ Department of Clinical Laboratory People’s Hospital of Baise Baise China; ^4^ Department of Laboratory Medicine The Affiliated Hospital of Guilin Medical University Guilin China; ^5^ Department of Dermatology The Affiliated Hospital of Youjiang Medical University for Nationalities Baise China

**Keywords:** LncRNA‐GAS5, miR‐21, PTEN, single nucleotide polymorphism, systemic lupus erythematosus

## Abstract

Growth arrest‐specific 5 (GAS5) is a kind of long non‐coding RNAs (lncRNAs). Previous studies showed that down‐regulation of LncRNA‐GAS5 was involved in the development of systemic lupus erythematosus (SLE). However, the regulatory mechanism of down‐expressed LncRNA‐GAS5 in SLE remains obscure. In this study, we aimed to investigate the association of LncRNA‐GAS5 polymorphism with SLE risk. And further explore how LncRNA‐GAS5 is involved in the occurrence of SLE. Here, we evaluated the relationship between the risk for the development of SLE and the 5‐base pair (AGGCA/‐) insertion/deletion (I/D) polymorphism (rs145204276) in the LncRNA‐GAS5 promoter region. A custom 36‐Plex SNPscan kit was used for genotyping the LncRNA‐GAS5 polymorphisms. The LncRNA‐GAS5 and miR‐21 target prediction was performed using bioinformatics software. Enzyme‐linked immunosorbent assay (ELISA) and quantitative real‐time PCR (qRT‐PCR) were performed to assess GAS5 and miR‐21 mRNA expression and PTEN protein expression. The results revealed that rs145204276 resulted in a decreased risk of SLE (DD genotypes vs II genotypes: adjusted OR = 0.538, 95% CI, 0.30‐0.97, *P* = .039; ID genotypes vs II genotypes: adjusted OR = 0.641, 95% CI, 0.46‐0.89, *P* = .007; ID/DD genotypes vs II genotypes: adjusted OR = 0.621, 95% CI, 0.46‐0.84, *P* = .002; D alleles vs I alleles: adjusted OR = 0.680, 95% CI, 0.53‐0.87, *P* = .002). A reduced incidence of renal disorders in SLE was found to be related to ID/DD genotypes and D alleles (ID/DD genotypes vs II genotypes: OR = 0.57, 95% CI, 0.36‐0.92, *P* = .020; D alleles vs I alleles: OR = 0.63, 95% CI, 0.43‐0.93, *P* = .019). However, no significant association of rs2235095, rs6790, rs2067079 and rs1951625 polymorphisms with SLE risk was observed (*P* > .05). Additionally, haplotype analysis showed that a decreased SLE risk resulted from the A‐A‐C‐G‐D haplotype (OR = 0.67, 95% CI, 0.49‐0.91, *P* = .010). Also, patients in the SLE group showed a down‐regulated expression of LncRNA‐GAS5 and PTEN than the healthy volunteers; however, patients with rs145204276 ID/DD genotypes showed up‐regulated expression of LncRNA‐GAS5 and PTEN compared with patients carrying the II genotype. Furthermore, the miR‐21 levels were considerably up‐regulated in the SLE group than the healthy volunteers, and patients with rs145204276 ID/DD genotype had lower miR‐21 levels than the ones with the II genotype. Thus, we found that the LncRNA‐GAS5/miR‐21/PTEN signalling pathway was involved in the development of SLE, where LncRNA‐GAS5 acted as an miR‐21 target, and miR‐21 regulated the expression of PTEN. These findings indicated that the rs145204276 ID/DD genotypes in the LncRNA‐GAS5 gene promoter region may be protected against SLE by up‐regulating the expression of LncRNA‐GAS5, which consecutively regulated miR‐21 and PTEN levels.

## 1 INTRODUCTION

Systemic lupus erythematosus (SLE) is a chronic autoimmune disorder that may damage multiple tissue/organs by generating autoantibodies and immune complexes.^1^, ^2^ It is a heterogeneous disease with diverse clinical characteristics.^3^ Most SLE patients exhibit a range of symptoms, including malar rash, nephritis, arthritis and neurologic disorders, which significantly reduce the quality of life.^4^, ^5^ Previous studies have shown that the development of SLE results from an imbalanced immune system, including aberrant excretion of cytokines, along with the modified immune cellular responses.^6^, ^7^, ^8^, ^9^, ^10^ The pathogenesis of SLE involves disorders related to genetics, immunity, environment and hormones; however, the precise mechanism of pathogenesis is unclear.^2^ Thus, Understanding the pathways of development of SLE would help in the identification of novel therapies.

LncRNAs are non‐protein coding transcripts that are > 200 nucleotides in length.^11^ Recent studies have shown that lncRNAs, which were previously considered as mere transcriptional noise, regulate gene expression at the transcriptional levels.^12^ The pathogenesis of several autoimmune inflammatory disorders, such as rheumatoid arthritis, SLE, multiple sclerosis, ulcerative colitis, hyperthyroidism and chronic liver disease, are known to involve lncRNAs.^13^, ^14^, ^15^ The aberrant expression of GAS5, a new type of lncRNAs, has been reported in SLE patients and animal models.^16^, ^17^ The lncRNA‐mRNA co‐expression analysis showed that LncRNA‐GAS5, lnc0640 and lnc5150 were involved in SLE pathogenesis through the mitogen‐activated protein kinase pathway (MAPK), and the LncRNA‐GAS5 in plasma could be used as SLE biomarkers.^16^ In addition, the chromosomal locus of LncRNA‐GAS5, 1q25, has been shown to be related with human SLE development in genetic studies.^18^

MicroRNAs (miRNAs) are non‐coding RNAs molecules, containing approximately 21‐23 nucleotides, which promote translational repression or degradation of their target mRNAs to regulate gene expression.^6^ miRNAs have been identified as novel biomarkers and potential therapeutic targets in several diseases, and they perform diverse immunoregulatory functions.^19^ Growing evidence has shown that the abnormal expression of miR‐21 is a contributor to SLE.^6^, ^20^ Here, we performed a bioinformatics analysis for target gene prediction and identified the probable binding site between miR‐21 and LncRNA‐GAS5. Furthermore, we identified the phosphatase and tensin homolog (PTEN) as the direct miR‐21target.

Several genome‐wide association studies (GWAS) have revealed the frequent use of single nucleotide polymorphisms (SNPs) as genetic markers.^21^ SNPs associated with lncRNAs are known to impact an individual's susceptibility to autoimmune diseases. For example, the lnc0640 gene rs13039216 TT genotype resulted in a reduced risk of rheumatoid arthritis (RA) and the G allele of rs141561256 in lnc5150 gene was significantly associated with the rheumatoid factor in RA patients.^22^ Furthermore, the variation at rs13259960 A>G, which weakened the STAT1 recruitment to the enhancer that looped to the SLEAR promoter, caused a reduced SLEAR expression in SLE patients.^23^ However, there are no published reports on the link between the LncRNA‐GAS5 gene polymorphisms and the SLE risk. This case‐control study investigated whether LncRNA‐GAS5 gene polymorphisms contributed to the development of SLE. Additionally, we assessed the impact of LncRNA‐GAS5 polymorphisms on LncRNA‐GAS5, miR‐21 and PTEN levels.

## 2 MATERIALS AND METHODS

### 2.1 Study subjects

We recruited 302 SLE patients (63 male and 239 female patients, average age 38.77 ± 14.41 years) and 396 age and gender‐matched healthy volunteers (106 male and 290 female volunteers, average age 39.85 ± 11.79 years) from the Affiliated Hospital of Youjiang Medical University for Nationalities and People's Hospital of Baise between June 2016 and September 2018. Written informed consent was obtained from all patients before study initiation. The study design was sanctioned by the ethics committee of the hospital. All patients met the SLE classification standards revised by the American rheumatology Society (ACR) in 1997.^24^ The clinical characteristics were collected based on medical records or questionnaires and were reviewed by senior doctors. We collected clinical data for diverse features, including malar rash, arthritis, leucopenia, renal disorder, thrombocytopenia, photosensitivity, neurological disorder, ribonucleoprotein antibody (Anti‐RNP), antinuclear antibody (ANA), smith antibody (Anti‐Sm), double‐stranded DNA antibody (Anti‐dsDNA), complement 3 (C3) and complement 4 (C4) antibodies. The healthy volunteers were unrelated and did not have any history of SLE, autoimmune diseases, cancer or other inflammatory diseases.

### 2.2 SNPs selection and LncRNA‐GAS5 genotyping

The selection criteria of SNPs were as follows: minor allele frequency (MAF) of LncRNA‐GAS5 SNPs should be >5% in the Han Chinese population of Beijing (CHB); searching for SNPs in the promoter region, exon region, 5′‐UTR and 3′‐UTR. Finally, we selected rs145204276 (promoter region), rs2235095 (intron region), rs6790 (exon region), rs2067079 (intron region) and rs1951625 (intron region) for further experimentation.

We isolated genomic DNA from peripheral blood samples and stored at −80°C. Online primer v3.0 was used for designing the PCR primers (http://primer3.ut.ee/) and synthesized by Shanghai Genesky Biotechnologies Inc (Table S1). The total volume of ligation reaction system was 20.0 μL, including 8.0 μL of sample DNA, 10.0 μL of 2× ligation buffer, 0.8 μL of DNA ligase, and 0.2 μL of connect probe mixture; the insufficient volume was supplemented with sterilized distilled water. Reaction parameters were as follows: 98°C 2 minutes; 95°C 30 seconds, 58°C 3 hours; 5 cycles; 94°C 2 minutes and 72°C forever. The total volume of multiplex PCR reaction system was 20 μL, including 1.0 μL of ligation product, 10.0 μL of 2× PCR buffer, 0.4 μL of TaqDNA polymerase, 0.5 μL of multiplex fluorescent amplification primer and 8.1 μL of sterilized distilled water. Multiple PCR reaction parameters were as follows: 95°C 2 minutes; 94°C 20 seconds, 62°C 40 seconds (each cycle reduced 0.5°C), 72°C 1.5 minutes, 9 cycles; 94°C 20 seconds, 57°C 40 seconds, 72°C 1.5 minutes, 25 cycles; 68°C 60 minutes and 4°C forever. The multiplex PCR product was diluted with 10 times of ddH_2_O, mixed with 1.0 μL of liz500 and 8.9 μL Hi‐Di, denatured at 95°C for 5 minutes. ABI3500 sequencer (ABI) was used for genotyping, and GeneMapper 4.1 software was used to analyse the original data. For quality control (QC), we randomly selected 5% of the samples for Sanger sequencing and found the results to be 100% accurate and consistent.

### 2.3 Extraction of RNA and quantitative real‐time polymerase chain reaction (qRT‐PCR)

We collected peripheral blood samples (3 mL) from each patient in an EDTA‐containing tube, followed by the isolation and purification of peripheral blood mononuclear cells (PBMCs) using the Ficoll‐Hypaque density gradient centrifugation. The TRIzol reagent was used for total RNA extraction from the PBMCs (Invitrogen), and a Hangzhou Nano‐300 spectrophotometer was used to measure the RNA concentrations.

The PrimeScript™ RT reagent Kit or Mir‐X™ miRNA First‐Strand Synthesis Kit (Takara) was used for cDNA synthesis based on the manufacturer's method. Next, qRT‐PCR was performed using SYBR Green (SYBR^®^ Premix Ex Taq™ II, Takara) on an Applied Biosystems 7500 Real‐Time PCR system. The qRT‐PCR reaction system contained about 2 µL of cDNA, forward and reverse primers 0.8 µL each, 10× ROX Reference Dye II 0.4 µL, 2× SYBR Premix Ex Taq II 10 µL, RNase Free dH_2_O 6 µL and a total volume of 20 µL. The RT‐PCR conditions were as follows: 95°C for 30 seconds; 40 cycles (95°C for 5 seconds and 62°C for 34 seconds). GAPDH or U6 were used as the internal references, and the 2^−ΔΔCt^ method was used to quantify the gene expression. The oligonucleotides used as primers were as follows: LncRNA‐GAS5 Forward Primer 5′‐ACACAGGCATTAGACAGAA‐3′, Reverse Primer 5′‐CCAGGAGCAGAACCATTA‐3′; miR‐21 Primer 5′‐CAACACCAGUCGAUGGGCUGU‐3′, universal downstream primers: HmiRQP0315 (Genecopoeia); GAPDH Forward Primer 5′‐GCCCAATACGACCAAATCC‐3′, Reverse Primer 5′‐AGCCACATCGCTCAG ACAC‐3′; U6‐Forward Primer 5′‐CTCGCTTCGGCAGCACA‐3′, Reverse Primer 5′‐CCGCTGGTTTCAT ATGGTG G‐3′. The melt curve analysis results for LncRNA‐GAS5 and miR‐21 expression analysis (Figure S1 and S2).

### 2.4 Serum PTEN determination

We obtained blood samples from both SLE patients and healthy volunteers. After clotting at room temperature, the serum was isolated and stored at −80°C for subsequent testing. The concentration of serum PTEN was measured using appropriate ELISA kits (Human PTEN ELISA Kit), and absorbance (OD value) was measured at 450 nm using an RT‐6000 enzyme micro‐plate reader. A standard calibration curve was used to determine the concentration of serum PTEN (Figure S3).

### 2.5 Statistical analysis

The SPSS software v17.0 was used for data analysis. The PASS software (version 15.0) was used to evaluate the sample size. A chi‐squared test was performed to evaluate whether the study population met the Hardy Weinberg equilibrium (HWE). Differences of age between control groups and SLE patients were compared by using Student's *t* test, whereas difference of gender was evaluated by using chi‐square test. Genotype and allele frequencies of GAS5 were compared by chi‐square test. Differences of GAS5, miR‐21 and PTEN expression levels were compared using Mann‐Whitney *U* test. The association between five SNPs polymorphisms and SLE risk was evaluated based on the odds ratio (OR) and 95% confidence interval (95% CI). Unconditional logistic regression was performed to adjust OR and 95% CI based on age and gender. Spearman's rank correlation analysis was used to analyse relationships between the expression levels of GAS5, miR‐21 and PTEN. The online SHEsis software was used for performing the haplotype analysis. The online bioinformatics software was used to predict the potential miR‐21 targets (http://mirtarbase.cuhk.edu.cn/php/search.php). A *P*‐value < .05 represented a statistically significant difference. Besides, we performed Bonferroni correction to eliminate the probability of false‐positive findings in the genetic model analyses, the *P*‐value < (.05/5) indicates statistical significance after Bonferroni correction.

## 3 RESULTS

### 3.1 Study subjects

Table 1 Lists the clinical characteristics of patients with SLE and healthy volunteers. There were insignificant differences regarding age and gender distribution between the SLE patient group and the healthy group (*P* > .05).

**TABLE 1:** Clinical characteristics of the SLE patients and controls

Characteristics	SLE patients (n = 302)	Control group (n = 396)	*P*‐value
Age, y (mean ± SD)	38.77 ± 14.41	39.85 ± 11.79	.291
Male/female (%)	63 (20.9)/239 (79.1)	106 (26.8)/290 (73.2)	.071
Malar rash, n (%)	155 (51.3)	–	–
Arthritis, n (%)	132 (43.7)	–	–
Renal disorder, n (%)	161 (53.3)	–	–
Photosensitivity, n (%)	141 (46.7)	–	–
Neurologic disorder, n (%)	22 (7.3)	–	–
ANA, n (%)	281 (93.0)	–	–
Anti‐RNP, n (%)	96 (31.8)	–	–
Anti‐dsDNA, n (%)	138 (45.7)	–	–
Anti‐Sm, n (%)	102 (33.7)	–	–
Leucopenia, n (%)	81 (26.8)	–	–
Thrombocytopenia, n (%)	49 (16.2)	–	–
Low levels of C3, n (%)	177 (58.6)	–	–
Low levels of C4, n (%)	150 (49.7)	–	–

Abbreviations: ANA, antinuclear antibody; Anti‐dsDNA, double‐stranded DNA antibody; Anti‐RNP, ribonucleoprotein antibody; Anti‐Sm, smith antibody; C3, complement 3; C4, complement 4; SLE, systemic lupus erythematosus.

### 3.2 Association of LncRNA‐GAS5 polymorphisms with SLE risk

**FIGURE 1:**
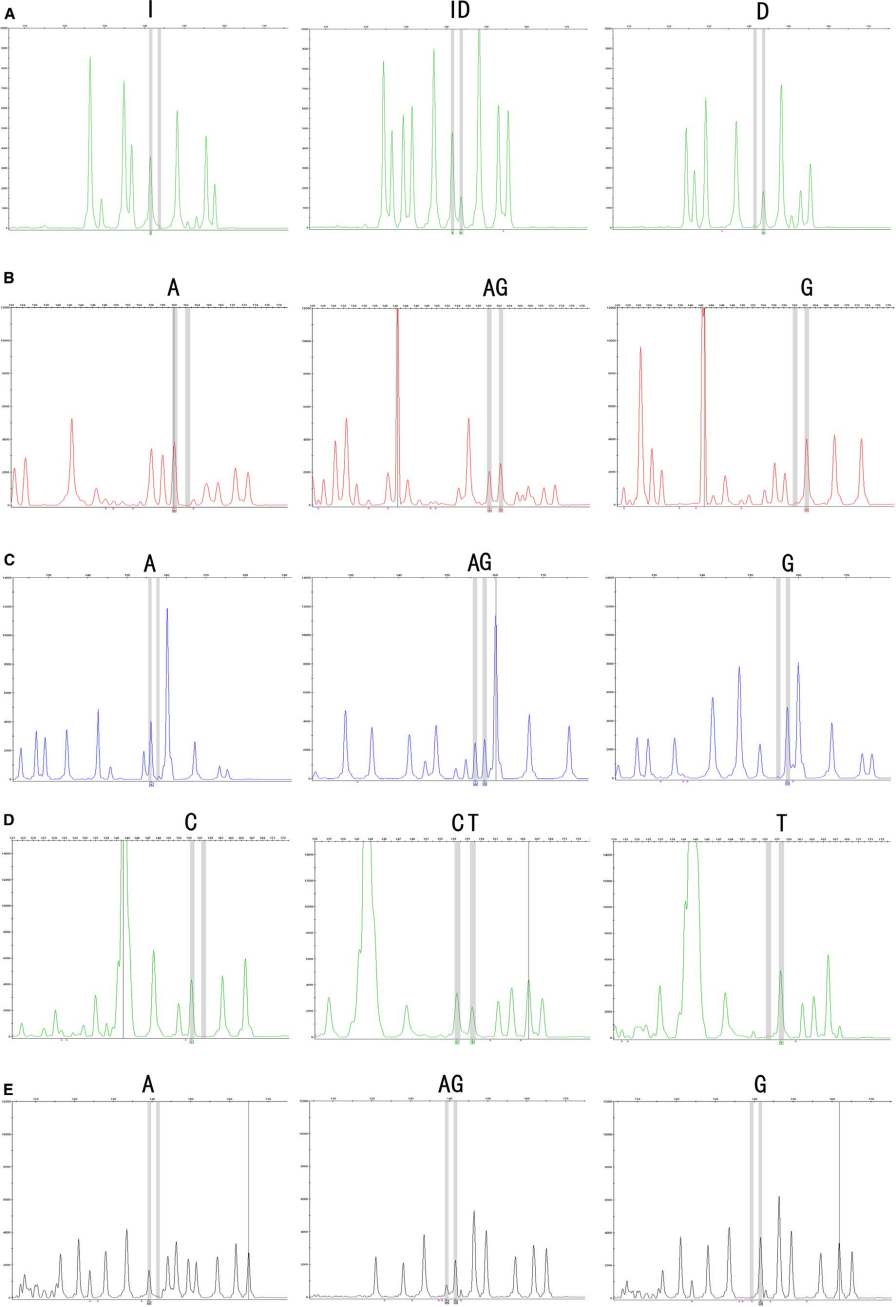
Fluorescence capillary electrophoresis map of genotypes for GAS5 gene polymorphisms. A, Capillary electrophoresis showed II, ID and DD genotypes for rs145204276; B, AA, AG and GG genotypes for rs2235095; C, AA, AG and GG genotypes for rs6790; D, CC, CT and TT genotypes for rs2067079; E, AA, AG and GG genotypes for rs1951625

We found three genotypes in rs145204276, rs2235095, rs6790, rs2067079 and rs1951625 polymorphisms (Figure 1). Table 2 summarizes the genotypes and the allele frequencies of these five SNPs of the LncRNA‐GAS5 gene in both SLE patients and healthy volunteers. The genotype distributions in the control group agreed with the HWE (*P* > .05). We found that the minor D allele of rs145204276 was related to a reduced risk of SLE than the major I allele (D alleles vs I alleles: adjusted OR = 0.680, 95% CI, 0.53‐0.87, *P* = .002). In addition, the DD genotype, ID genotype and the Dominant model resulted in a decreased risk of SLE (DD genotypes vs II genotypes: adjusted OR = 0.538, 95% CI, 0.30‐0.97, *P* = .039; ID genotypes vs II genotypes: adjusted OR = 0.641, 95% CI, 0.46‐0.89, *P* = .007; ID/DD genotypes vs II genotypes: adjusted OR = 0.621, 95% CI, 0.46‐0.84, *P* = .002). However, no significant association of rs2235095, rs6790, rs2067079 and rs1951625 polymorphisms with SLE risk was observed (*P* > .05). After Bonferroni correction, no significant association between the rs145204276 DD genotype and the risk of SLE was observed, the significance of multiplex detection at the *P* < .01 (0.05/5) level was retained for rs145204276 ID genotype, D allele and dominant model.

**TABLE 2:** Association between LncRNA‐GAS5 polymorphisms and risk of SLE

Polymorphism	Controls n = 396 (%)	HWE, *P* ^a^	SLE patients, n = 302 (%)	HWE *P* ^b^	OR (95% CI)	Adjusted OR (95% CI)^a^	*P*	Adjusted *P* ^c^
rs145204276		.712		.194				
II	196 (49.5)		186 (61.6)		1.00 (Ref)	1.00 (Ref)		
ID	163 (41.2)		97 (32.1)		0.627 (0.46‐0.87)	0.641 (0.46‐0.89)	.004	.007*
DD	37 (9.3)		19 (6.3)		0.541 (0.30‐0.98)	0.538 (0.30‐0.97)	.041	.039
I	555 (70.1)		469 (77.6)		1.00 (Ref)	1.00 (Ref)		
D	237 (29.9)		135 (22.4)		0.674 (0.53‐0.86)	0.680 (0.53‐0.87)	.002	.002*
Dominant model								
II	196 (49.5)		186 (61.6)		1.00 (Ref)	1.00 (Ref)		
ID/DD	200 (50.5)		116 (38.4)		0.611 (0.45‐0.83)	0.621 (0.46‐0.84)	.002	.002*
Recessive model								
II/ID	359 (90.7)		283 (93.7)		1.00 (Ref)	1.00 (Ref)		
DD	37 (9.3)		19 (6.3)		0.651 (0.37‐1.16)	0.641 (0.36‐1.14)	.144	.131
rs2235095		.664		.194				
GG	135 (34.1)		113 (37.4)		1.00 (Ref)	1.00 (Ref)		
AG	196 (49.5)		152 (50.3)		0.926 (0.67‐1.29)	0.937 (0.67‐1.30)	.648	.700
AA	65 (16.4)		37 (12.3)		0.680 (0.42‐1.09)	0.688 (0.43‐1.11)	.111	.125
G	466 (58.8)		378 (62.6)		1.00 (Ref)	1.00 (Ref)		
A	326 (41.2)		226 (37.4)		0.855 (0.69‐1.06)	0.861 (0.69‐1.07)	.156	.177
Dominant model								
GG	135 (34.1)		113 (37.4)		1.00 (Ref)	1.00 (Ref)		
AG/AA	261 (65.9)		189 (62.6)		0.865 (0.63‐1.18)	0.875 (0.64‐1.20)	.363	.404
Recessive model								
GG/AG	331 (83.6)		265 (87.7)		1.00 (Ref)	1.00 (Ref)		
AA	65 (16.4)		37 (12.3)		0.711 (0.46‐1.10)	0.715 (0.46‐1.11)	.124	.132
rs1951625		.781		.380				
GG	212 (53.5)		164 (54.3)		1.00 (Ref)	1.00 (Ref)		
AG	154 (38.9)		113 (37.4)		0.949 (0.69‐1.30)	0.946 (0.69‐1.30)	.744	.733
AA	30 (7.6)		25 (8.3)		1.077 (0.61‐1.90)	1.053 (0.60‐1.86)	.798	.860
G	578 (73.0)		441 (73.0)		1.00 (Ref)	1.00 (Ref)		
A	214 (27.0)		163 (27.0)		0.998 (0.79‐1.27)	0.991 (0.78‐1.26)	.989	.938
Dominant model								
GG	212 (53.5)		164 (54.3)		1.00 (Ref)	1.00 (Ref)		
AG/AA	184 (46.5)		138 (45.7)		0.970 (0.72‐1.31)	0.964 (0.71‐1.30)	.840	.810
Recessive model								
GG/AG	366 (92.4)		277 (91.7)		1.00 (Ref)	1.00 (Ref)		
AA	30 (7.6)		25 (8.3)		1.101 (0.63‐1.92)	1.077 (0.62‐1.88)	.733	.792
rs2067079		.305		.163				
CC	250 (63.1)		197 (65.2)		1.00 (Ref)	1.00 (Ref)		
CT	125 (31.6)		89 (29.5)		0.904 (0.65‐1.26)	0.899 (0.65‐1.25)	.547	.530
TT	21 (5.3)		16 (5.3)		0.967 (0.49‐1.90)	0.962 (0.49‐1.90)	.922	.911
C	625 (78.9)		483 (80.0)		1.00 (Ref)	1.00 (Ref)		
T	167 (21.1)		121 (20.0)		0.938 (0.72‐1.22)	0.934 (0.72‐1.22)	.630	.611
Dominant model								
CC	250 (63.1)		197 (65.2)		1.00 (Ref)	1.00 (Ref)		
CT/TT	146 (36.9)		105 (34.8)		0.913 (0.67‐1.25)	0.908 (0.66‐1.24)	.567	.548
Recessive model								
CC/CT	375 (94.7)		286 (94.7)		1.00 (Ref)	1.00 (Ref)		
TT	21 (5.3)		16 (5.3)		0.999 (0.51‐1.95)	0.995 (0.51‐1.95)	.998	.989
rs6790		.827		.125				
GG	142 (35.9)		107 (35.4)		1.00 (Ref)	1.00 (Ref)		
AG	192 (48.5)		156 (51.7)		1.078 (0.78‐1.50)	1.076 (0.77‐1.50)	.652	.662
AA	62 (15.6)		39 (12.9)		0.835 (0.52‐1.34)	0.851 (0.53‐1.37)	.454	.505
G	476 (60.1)		370 (61.3)		1.00 (Ref)	1.00 (Ref)		
A	316 (39.9)		234 (38.7)		0.938 (0.72‐1.22)	0.934 (0.72‐1.22)	.630	.611
Dominant model								
GG	142 (35.9)		107 (35.4)		1.00 (Ref)	1.00 (Ref)		
AG/AA	254 (64.1)		195 (64.6)		1.019 (0.75‐1.39)	1.021 (0.75‐1.40)	.907	.895
Recessive model								
GG/AG	334 (84.3)		263 (87.1)		1.00 (Ref)	1.00 (Ref)		
AA	62 (5.6)		39 (12.9)		0.799 (0.52‐1.23)	0.815 (0.53‐1.26)	.308	.357

Abbreviation: HWE, Hardy‐Weinberg equilibrium.

^a^ Control group.

^b^ SLE patients group, OR, odds ratio; CI, confidence interval; Ref, reference.

^c^ Adjusted by age and gender. The P* value indicates statistical significance after Bonferroni correction.

### 3.3 Association of rs145204276 polymorphism with clinical features

We further stratified the distribution of rs145204276 polymorphism in thirteen specific clinical features in both SLE positive and negative patients (Table 3). The results revealed a negative correlation between a reduced risk of renal disorders in SLE and the ID/DD genotypes and D allele (ID/DD genotypes vs II genotypes: OR = 0.57, 95% CI, 0.36‐0.92, *P* = .020; D alleles vs I alleles: OR = 0.63, 95% CI, 0.43‐0.93, *P* = .019).

**TABLE 3:** Association between genotypes and alleles frequencies in rs145204276 with clinical features in SLE patients

Clinical features	+/−	Genotypes (%)		*P*	OR, ID + DD vs II	Alleles (%)		*P*	OR, D vs I
		II	ID + DD			I	D		
Malar rash	+	92 (59.4)	63 (40.6)	.412	1.22 (0.76‐1.93)	234 (75.5)	76 (24.5)	.190	1.29 (0.88‐1.90)
	−	94 (63.9)	53 (36.1)			235 (79.9)	59 (20.1)		
Arthritis	+	82 (62.1)	50 (37.9)	.867	0.96 (0.60‐1.53)	202 (76.5)	62 (23.5)	.556	0.12 (0.76‐1.65)
	−	104 (61.2)	66 (38.8)			267 (78.5)	73 (21.5)		
Renal disorder	+	109 (67.7)	52 (32.3)	.020	0.57 (0.36‐0.92)	262 (81.4)	60 (18.6)	.019	0.63 (0.43‐0.93)
	−	77 (54.6)	64 (45.4)			207 (73.4)	75 (26.6)		
Photosensitivity	+	81 (57.4)	60 (42.6)	.166	1.39 (0.87‐2.21)	211 (74.8)	71 (25.2)	.119	1.36 (0.92‐1.99)
	−	105 (65.2)	56 (34.8)			258 (80.1)	64 (19.9)		
Neurologic disorder	+	12 (54.5)	10 (45.5)	.481	1.37 (0.57‐3.28)	32 (72.7)	12 (27.3)	.416	1.33 (0.67‐2.66)
	−	174 (62.1)	106 (37.9)			437 (78.0)	123 (22.0)		
ANA	+	174 (61.9)	107 (38.1)	.664	0.82 (0.33‐2.01)	438 (77.9)	124 (22.1)	.536	0.80 (0.39‐1.63)
	−	12 (57.1)	9 (42.9)			31 (73.8)	11 (26.2)		
Anti‐RNP	+	54 (56.3)	42 (43.8)	.193	1.39 (0.85‐2.27)	143 (74.5)	49 (25.5)	.202	1.30 (0.87‐1.94)
	−	132 (64.1)	74 (35.9)			326 (79.1)	86 (20.9)		
Anti‐dsDNA	+	86 (62.3)	52 (37.7)	.811	0.95 (0.59‐1.51)	213 (77.2)	63 (22.8)	.797	1.05 (0.72‐1.54)
	−	100 (61.0)	64 (39.0)			256 (78.0)	72 (22.0)		
Anti‐Sm	+	67 (65.7)	35 (34.3)	.296	0.77 (0.47‐1.26)	165 (80.9)	39 (19.1)	.173	0.75 (0.49‐1.14)
	−	119 (59.5)	81 (40.5)			304 (76.0)	96 (24.0)		
Leucopenia	+	51 (63.0)	30 (37.0)	.766	0.92 (0.55‐1.56)	125 (77.2)	37 (22.8)	.861	1.04 (0.68‐1.60)
	−	135 (61.1)	86 (38.9)			344 (77.8)	98 (22.2)		
Thrombocytopenia	+	25 (51.0)	24 (49.0)	.097	1.68 (0.91‐3.11)	70 (71.4)	28 (28.6)	.106	1.49 (0.92‐2.43)
	−	161 (63.6)	92 (36.4)			399 (78.9)	107 (21.1)		
Low levels of C3	+	115 (65.0)	62 (35.0)	.150	0.71 (0.44‐1.13)	283 (79.9)	71 (20.1)	.107	0.73 (0.50‐1.07)
	−	71 (56.8)	54 (43.2)			186 (74.4)	64 (25.6)		
Low levels of C4	+	96 (64.0)	54 (36.0)	.392	0.82 (0.51‐1.30)	235 (78.3)	65 (21.7)	.688	0.93 (0.63‐1.36)
	−	90 (59.2)	62 (40.8)			234 (77.0)	70 (23.0)		

### 3.4 Haplotype analysis of the LncRNA‐GAS5 polymorphisms with risk of SLE

Next, we assessed the haplotype frequencies of the five SNPs in the LncRNA‐GAS5 gene in both the SLE patients and the healthy volunteers using the online SHEsis software (Table 4). Approximately, 32.1% and 28.3% of the maximum haplotype (G‐G‐C‐G‐I) were observed in SLE patients and the healthy volunteers, respectively. We also found that reduced risk for SLE was related to the presence of the haplotype (A‐A‐C‐G‐D) (OR = 0.67, 95% CI, 0.49‐0.91, *P* = .010). After Bonferroni correction, the significant association between the haplotype (A‐A‐C‐G‐D) and the risk of SLE was observed.

**TABLE 4:** Haplotype analysis of the LncRNA‐GAS5 polymorphisms with risk of SLE

rs2235095	rs6790	rs2067079	rs1951625	rs145204276	Control group (2n = 792) (%)	SLE patients (2n = 604) (%)	OR (95% CI)	*P*
G	G	C	G	I	224 (28.3)	194 (32.1)	1.27 (0.99‐1.62)	.056
A	A	C	G	D	143 (18.1)	77 (12.8)	0.67 (0.49‐0.91)	.010*
G	G	T	A	I	118 (14.9)	92 (15.2)	1.06 (0.78‐1.43)	.704
A	A	C	G	I	100 (12.6)	74 (12.3)	0.99 (0.72‐1.38)	.978
G	G	C	A	I	31 (3.9)	13 (2.2)	0.56 (0.29‐1.08)	.080
G	G	C	G	D	9 (1.5)	24 (3.0)	0.52 (0.24‐1.11)	.085

Note

The P* value indicates statistical significance after Bonferroni correction.

Abbreviations: CI, confidence interval; OR, odds ratio.

### 3.5 Target gene prediction and LncRNA‐GAS5, miR‐21 and PTEN expression

**FIGURE 2:**
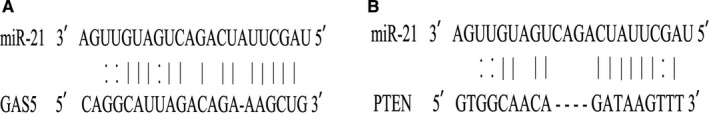
Bioinformatics software was used to predict target of miR‐21. A, Potential miR‐21 base pairing with GAS5; B, Putative binding site of miR‐21 in PTEN

**FIGURE 3:**
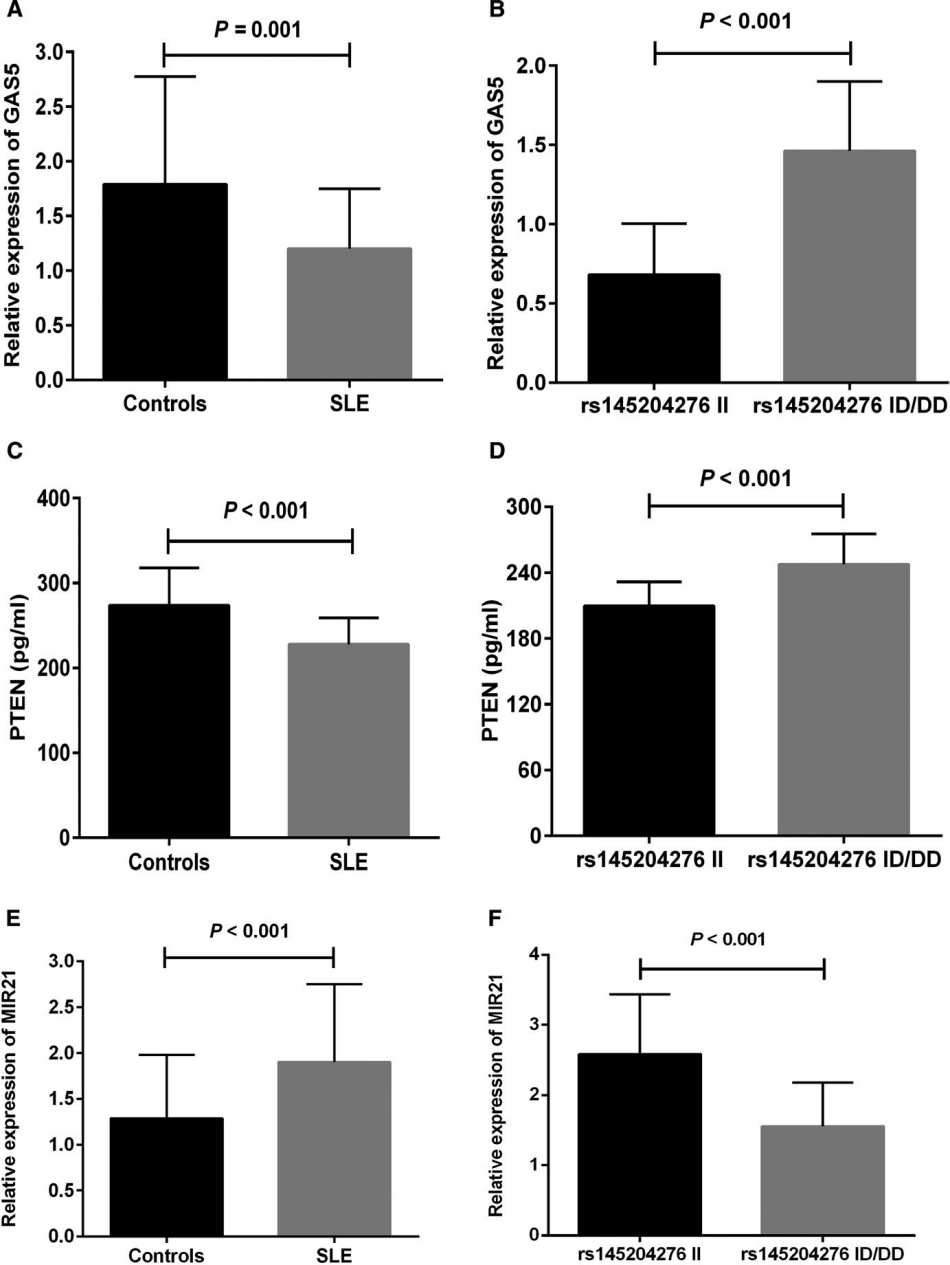
Relative expression of GAS5 in controls (n = 45) and SLE patients (n = 45), expression levels were normalized to GAPDH. A, Decreased level of GAS5 in SLE patients compared with controls (*P* = .001); B, Increased level of GAS5 in SLE patients carrying the rs145204276 ID + DD genotypes (n = 30) compared with those carrying the rs145204276 II genotype (n = 15) (*P* < .001). ELISA detection of PTEN expression. C, Serum level of PTEN in SLE patients (n = 90) and controls (n = 90), *P* < .001; D, Increased level of PTEN in SLE patients carrying the rs145204276 ID + DD genotypes (n = 43) compared with those carrying the rs145204276 II genotype (n = 47) (*P* < .001). The relative expression of miR‐21 in SLE patients (n = 45) and controls (n = 45), expression levels were normalized to U6. E, Increased level of miR‐21 in SLE patients compared with controls (*P* < .001); F, Decreased level of miR‐21 in SLE patients carrying the rs145204276 ID + DD genotypes (n = 30) compared with those carrying the rs145204276 II genotype (n = 15) (*P* < .001)

The potential miR‐21 targets were predicted through an online bioinformatics software. We identified that there were several base pair binding sites between miR‐21 and LncRNA‐GAS5 (Figure 2A). We further identified PTEN as the miR‐21 target protein (Figure 2B). Next, qRT‐PCR and ELISA were performed to compare LncRNA‐GAS5, miR‐21 and PTEN levels in SLE patients having different rs145204276 genotypes to explore the molecular mechanism between rs145204276 polymorphism and the development of SLE. SLE patients showed considerably reduced LncRNA‐GAS5 levels than the healthy volunteers (*P* = .001; Figure 3A). We further analysed whether rs145204276 genetic polymorphism could affect the expression level of the LncRNA‐GAS5 gene in SLE patients. We found higher LncRNA‐GAS5 levels in the rs145204276 ID/DD genotypes than the rs145204276 II genotype (*P* < .001; Figure 3B). Additionally, PTEN expression followed a trend similar to that of LncRNA‐GAS5, that is SLE patients showed a substantially reduced PTEN expression than the healthy volunteers (*P* < .001; Figure 3C) and the rs145204276 ID/DD genotype had elevated PTEN levels than the rs145204276 II genotype (*P* < .001; Figure 3D). On the contrary, in SLE patients, we observed significantly up‐regulated miR‐21 expression compared with the healthy volunteers (*P* < .001; Figure 3E). Furthermore, rs145204276 ID/DD genotypes had lower miR‐21 levels compared with the rs145204276 II genotype (*P* < .001; Figure 3F). The expression levels of GAS5, miR‐21 and PTEN in the controls and SLE patients were not significantly different between male and female (*P* > .05; Figure S4). In addition, spearman's rank correlation analysis showed that there was a correlation between the expression levels of GAS5, miR‐21 and PTEN (Table S2).

## 4 DISCUSSION

This study assessed the relationship between the risk associated with the development of SLE and the five SNPs (rs145204276, rs2235095, rs6790, rs2067079 and rs1951625) in the LncRNA‐GAS5 gene. This study is the first report that identified the allele and genotype frequencies of five SNPs in the LncRNA‐GAS5 gene and the relative risk of development of SLE in a subset of the Chinese population. We found that reduced risk for SLE was significantly related to the rs145204276 D allele, ID genotype, DD genotype and the dominant model (ID + DD genotypes vs II genotypes). ID + DD genotypes and D allele in rs145204276 resulted in a substantially reduced risk of renal disorder in SLE. Haplotype analysis found that the A‐A‐C‐G‐D haplotype was related to a reduced risk of SLE. However, no significant association of rs2235095, rs6790, rs2067079 and rs1951625 polymorphisms with SLE risk was observed (*P* > .05). Additionally, SLE patients had significantly down‐regulated LncRNA‐GAS5 and PTEN levels compared with the healthy volunteers; however, patients with rs145204276 ID/DD genotypes exhibited higher LncRNA‐GAS5 and PTEN levels than the patients with the II genotype. Also, SLE patients had considerably elevated miR‐21 levels than the healthy volunteers, and the patients with rs145204276 ID/DD genotype had lower miR‐21 levels than the ones with the II genotype. Thus, these findings suggested that the susceptibility to SLE was related to the rs145204276 polymorphism.

LncRNAs play multiple biological roles, including gene transcription, RNA splicing, protein transport and chromatin remodelling.^25^ Previous studies have revealed that lncRNAs are involved in the development and immune response of immune cells through different mechanisms and regulate the gene expression through multi‐functional interaction with DNA, RNA or protein.^26^, ^27^ LncRNA‐GAS5, a novel lncRNA that was identified in mouse NIH3T3 cells, localized at 1q25 and contains several small nucleolar RNAs.^28^, ^29^ Previous studies have shown that LncRNA‐GAS5 acts as a tumour suppressor for several types of cancers, such as liver cancer, breast cancer, colorectal cancer and prostate cancer.^30^, ^31^, ^32^, ^33^ SNP in the known protein‐coding genes may affect the expression or functioning of those genes through different mechanisms. Tao et al^34^ suggested that rs145204276 affected the transcriptional activity of LncRNA‐GAS5 and regulated its expression through CpG island methylation in the promoter region, which is linked with an increased susceptibility and progression of liver cancer. Zhu et al^35^ showed that rs55829688 CC + rs145204276 del/del genotype had an impact on the prognosis and survival rate of prostate cancer patients through the regulation of miR‐21/miR‐1284 expression, which consequently affected PDCD4, PTEN and AKT expression. In addition, rs145204276 del allele was linked with a significantly lower incidence of gastric cancer progression and metastasis.^36^ For several years, LncRNA‐GAS5 was regarded as a tumour suppressor gene. However, recent studies have shown that LncRNA‐GAS5 plays a role in the pathogenesis of immune‐mediated inflammatory diseases, such as SLE, multiple sclerosis, rheumatoid arthritis, inflammatory bowel disease and immune thrombocytopenia.^16^, ^37^, ^38^, ^39^, ^40^ To date, there are no reports indicating an association between LncRNA‐GAS5 gene polymorphism and the risk for SLE. We deduced that rs145204276 polymorphism in the promoter region had an impact on LncRNA‐GAS5 expression. Subsequently, we detected the level of expression of LncRNA‐GAS5 and compared LncRNA‐GAS5 expression with rs145204276 polymorphism. Here, the SLE patients had significantly down‐regulated LncRNA‐GAS5 levels in PBMCs compared with the healthy volunteers. Interestingly, Wu et al had also identified reduced plasma levels of LncRNA‐GAS5 in SLE patients compared with healthy volunteers.^16^ Additionally, elevated LncRNA‐GAS5 levels were observed in patients with rs145204276 ID + DD genotype than those with II genotype. LncRNA‐GAS5 gene rs145204276 polymorphism resulted in a decreased SLE risk, which could act as a protective factor. These outcomes support our hypothesis.

With the development of molecular biology technology and the advancement of genetics research. It has become increasingly clear that numerous miRNA response elements (MRE) exist on a wide variety of RNA transcripts, leading to the hypothesis that all RNA transcripts that contain miRNA MRE can communicate and regulate each other by competing specifically miRNAs, thus acting as competing endogenous RNAs (ceRNAs), it includes mRNA, lncRNA and circRNA. miRNA is a type of non‐coding RNA with a length of about 22 nt, which is the core molecule in the ceRNA hypothesis. It can promote the degradation of mRNAs or inhibit translation by binding with the 3′‐untranslated region (3′‐UTR) of target mRNA and further regulate a variety of biological behaviours.^41^ Yan et al^42^ found that lncRNA HIX003209 did not directly bind to TLR2, TLR4 and NF‐κB; however, it promoted TLR4/NF‐κB expression in macrophages through the sponge miR‐6089, which was critical for the development of rheumatoid arthritis. Additionally, Deng et al^43^ reported that the THRIL/miR‐34a/MCP‐1 axis played a key role in the development of SLE. Here, we performed bioinformatics analyses to identify the putative LncRNA‐GAS5 and PTEN binding sites in miR‐21. Subsequently, qRT‐PCR and ELISA were used to elucidate the regulatory relationship between LncRNA‐GAS5 and miR‐21/PTEN. SLE patients with rs145204276 ID/DD genotype have high expression of LncRNA‐GAS5 and low expression of miR‐21 in PBMC, which up‐regulates the expression level of PTEN in serum; on the contrary, SLE patients with rs145204276 II genotype have low expression of LncRNA‐GAS5, high level of miR‐21 and down‐regulate PTEN expression level in serum. A previous study had shown that PTEN was a direct target of miR‐21 and contributed to the effects exerted by miR‐21 on cell invasion.^44^ MiR‐21 regulated PTEN expression through 3′‐UTR, thus getting involved in the process of tumorigenesis in a wide range of cancers.^45^ LncRNAs act as miRNA sponges to restore the target gene expression via competitive regulatory interactions between lncRNAs, miRNAs and mRNAs. In this network, any abnormal expression of non‐coding RNA would contribute to the occurrence and development of diseases.

This study showed that LncRNA‐GAS5 gene polymorphisms played a critical role in the aetiology of SLE; however, it had the following limitations. First, due to the limited time and funds, the study sample size was relatively small, which probably restricted the reliability of relevant statistical tests. Second, all participants were recruited from the same hospital; thus, there exists a strong possibility of selection bias. Third, previous studies have shown that SLE results from complex interactions between genetic, environmental and hormonal factors. However, in our current study, due to the lack of relevant data, it was impossible to estimate the impact of gene‐environment interaction. Therefore, further studies will be performed with a large sample size and different genetic backgrounds, more functional experiments combined with animal research, which will enhance our understanding of the molecular interaction involved in this field more clearly and draw more powerful conclusions.

Thus, we demonstrated that the rs145204276 ID/DD genotypes in the promoter region of the LncRNA‐GAS5 gene acted as a protective factor towards the development of SLE, most probably by elevating LncRNA‐GAS5 expression. Additionally, LncRNA‐GAS5 may contribute to SLE in the pathogenesis by targeting PTEN through competitive binding to miR‐21. Thus, our findings provide a new direction to further our understanding of the pathogenesis mechanism and the role of LncRNA‐GAS5 in SLE. In the future, a larger sample size and different ethnic groups will be studied to confirm these findings.

## CONFLICT OF INTEREST

The authors declare no conflict of interests.

## AUTHOR CONTRIBUTIONS

**Ye‐Sheng Wei:** Funding acquisition (equal); Resources (equal); Validation (equal); Visualization (equal); Writing‐original draft (equal); Writing‐review & editing (equal). **Chun‐Hong Liu:** Conceptualization (equal); Data curation (equal); Formal analysis (equal); Methodology (equal); Project administration (equal); Resources (equal); Software (equal); Supervision (equal); Writing‐original draft (equal); Writing‐review & editing (equal). **Yu‐Lan Lu:** Data curation (equal); Formal analysis (equal); Funding acquisition (equal); Software (equal). **Hua‐Tuo Huang:** Methodology (equal). **Chun‐Fang Wang:** Formal analysis (equal); Project administration (equal); Resources (equal); Software (equal). **Hong‐Cheng Luo:** Data curation (equal); Formal analysis (equal). **Gui‐Jiang Wei:** Methodology (equal); Project administration (equal); Resources (equal); Software (equal). **Ming Lei:** Data curation (equal); Formal analysis (equal); Methodology (equal); Project administration (equal); Resources (equal). **Tan Tan:** Investigation (equal); Methodology (equal); Project administration (equal); Resources (equal); Software (equal); Writing‐original draft (equal). **Yan Wang:** Data curation (equal); Software (equal). **Yan‐Yun Huang:** Formal analysis (equal). **Yan Lan:** Conceptualization (equal); Funding acquisition (equal); Methodology (equal); Resources (equal); Writing‐original draft (equal); Writing‐review & editing (equal).

## Supporting information

Supplementary Material

## Data Availability

All data generated or analyzed during this study are included in this article.
