# Supplementary material for: Association of LncRNA‐GAS5 gene polymorphisms and PBMC LncRNA‐GAS5 level with risk of systemic lupus erythematosus in Chinese population
Source: J Cell Mol Med. 2021 Mar 16;25(7):3548–59. doi: 10.1111/jcmm.16438 (PMC8034459; doi:10.1111/jcmm.16438)
Supplement: Supplementary file 1 — Supplementary Material [file JCMM-25-3548-s001.docx]

**Supplementary Table 1. The primer sequences for LncRNA-GAS5** **genotyping.**

| **SNPs** | **Lengthh** | **Primer sequence** |
| --- | --- | --- |
| rs2235095 |  |  |
| AF | 36 | AGCTCAATCAATTAACGTTAACATCAATAAAACCTA |
| GF | 36 | AGCTCAATCAATTAACGTTAACATCAATAAAACTTG |
| 3F | 31 | TACCTTTAAAAGGTATGACAGGAACTRTCTT |
| rs6790 |  |  |
| AR | 28 | TGACTTGCTTGGGTAAGGACATGAACAT |
| GR | 28 | TGACTTGCTTGGGTAAGGACATGAACAC |
| 3R | 31 | AGTTCCTGTCATACCTTTTAAAGGTAYATGT |
| rs2067079 |  |  |
| CF | 36 | CAAACTTTCTTATTAATCATAACAAGACAAGAAACC |
| TF | 36 | CAAACTTTCTTATTAATCATAACAAGACAAGAAGCT |
| 3F | 28 | GCCATTTATTTAATRCATTCAGCACTAG |
| rs1951625 |  |  |
| AR | 21 | GCTGGAATGCAGTGGCTCCAT |
| GR | 21 | GCTGGAATGCAGTGGCTCTAC |
| 3R | 21 | ATCGGTTAATGGCAACCTCCG |
| rs145204276 |  |  |
| IF | 20 | AGGGGAGGGGGCGCGAGTCA |
| DF | 20 | CAGAGAGGGGAGGGGGCTCG |
| 3F | 20 | AGGAAAGCTCTGGGGATGGG |

Note: SNP, Single nucleotide polymorphisms; 3, 3' universal primer; F, forward; R, reverse.

**Supplementary Table 2. Correlation of** **expression levels of** **GAS5, miR-21 and PTEN**

| Gene | R | *P* |
| --- | --- | --- |
| GAS5-II vs. miR-21 | -0.611 | 0.016 |
| GAS5-II vs. PTEN | 0.864 | <0.001 |
| miR-21 vs. PTEN | -0.779 | 0.001 |
| GAS5-ID/DD vs. miR-21 | -0.704 | 0.003 |
| GAS5-ID/DD vs. PTEN | 0.891 | <0.001 |

**Supplementary Figure**

**
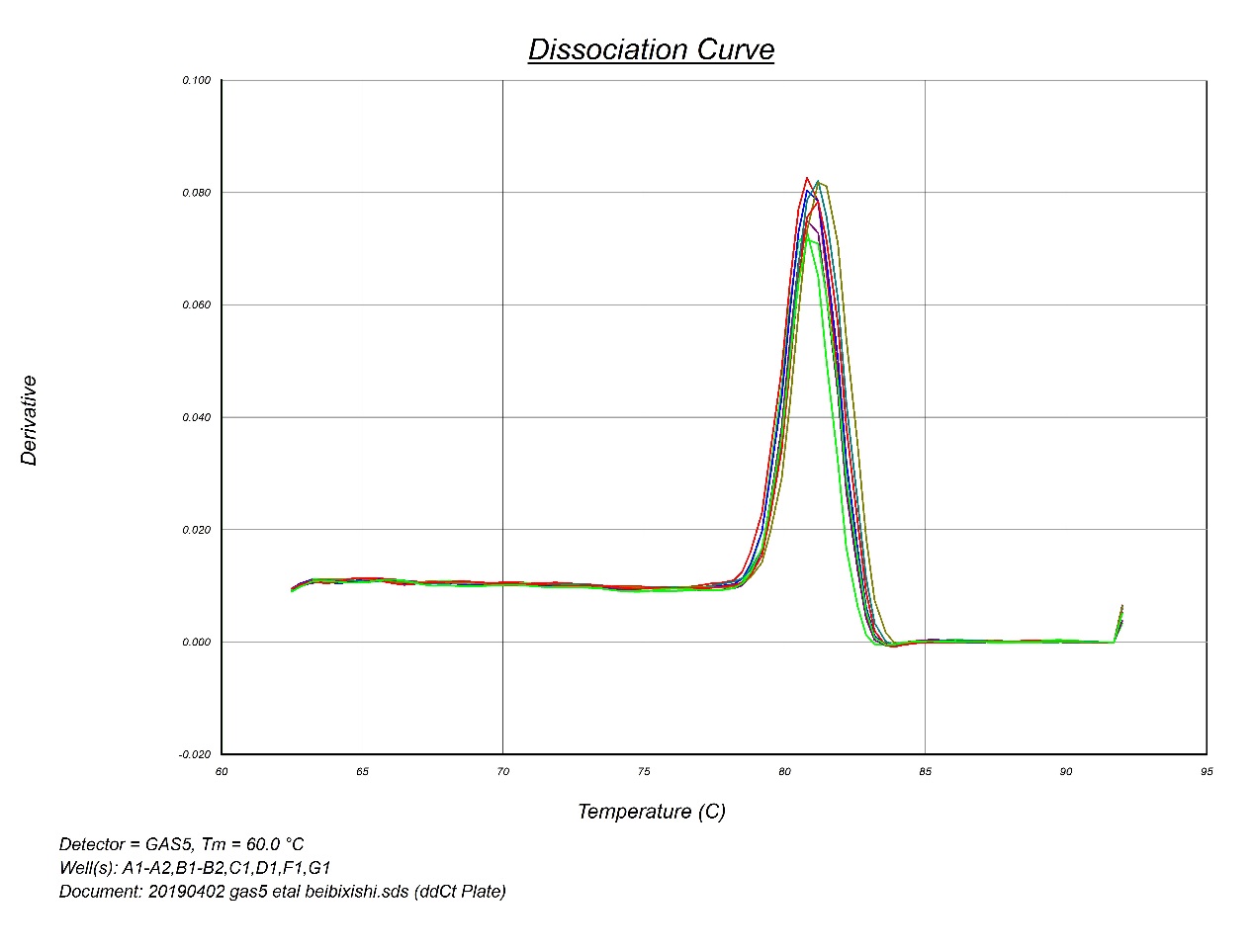
**

**Supplementary Figure 1.** The melt curve analysis results for the LncRNA-GAS5 expression analysis.


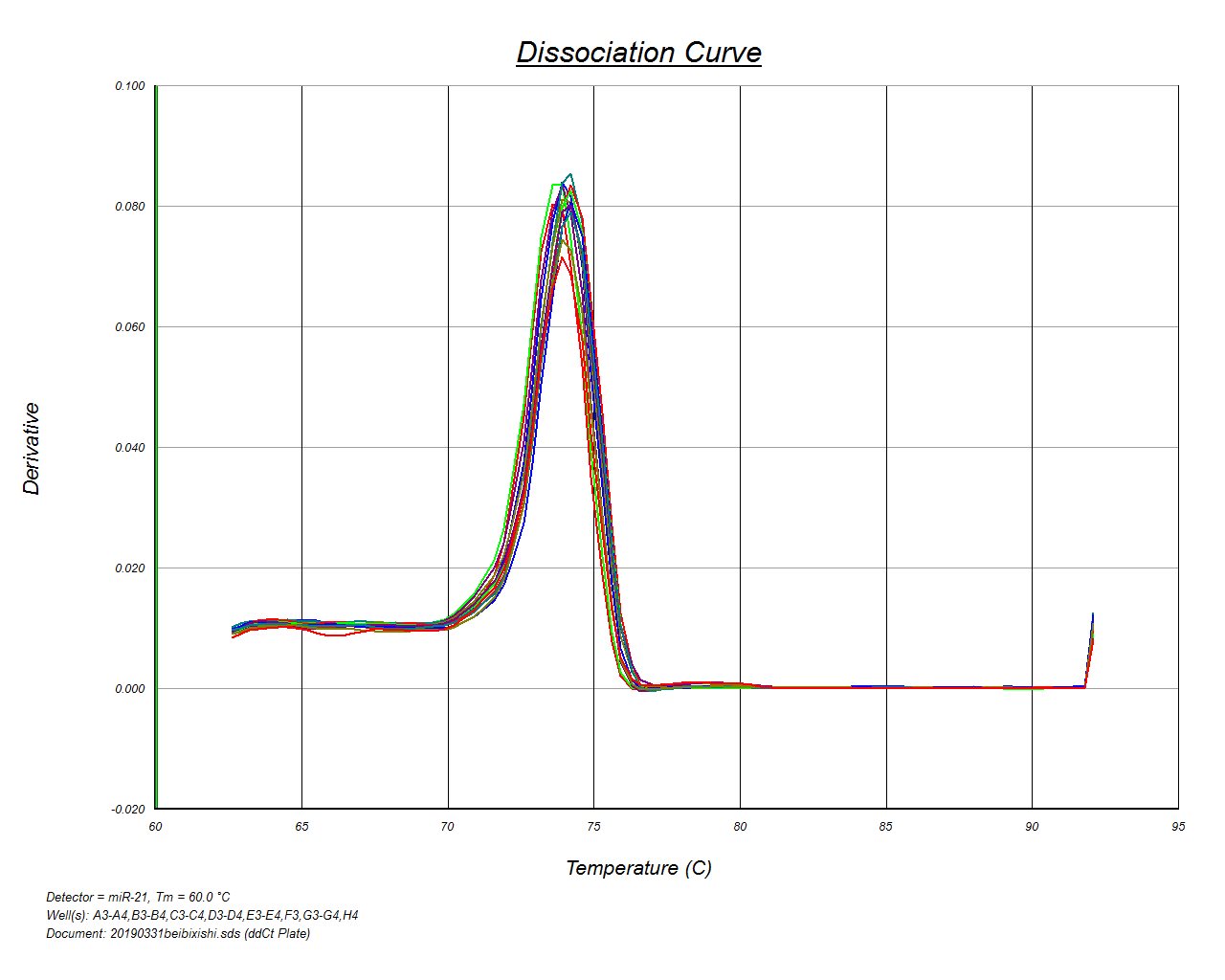


**Supplementary Figure 2.** The melt curve analysis results for the miR-21 expression analysis.

**Supplementary Figure 3.** The standard curve of PTEN.


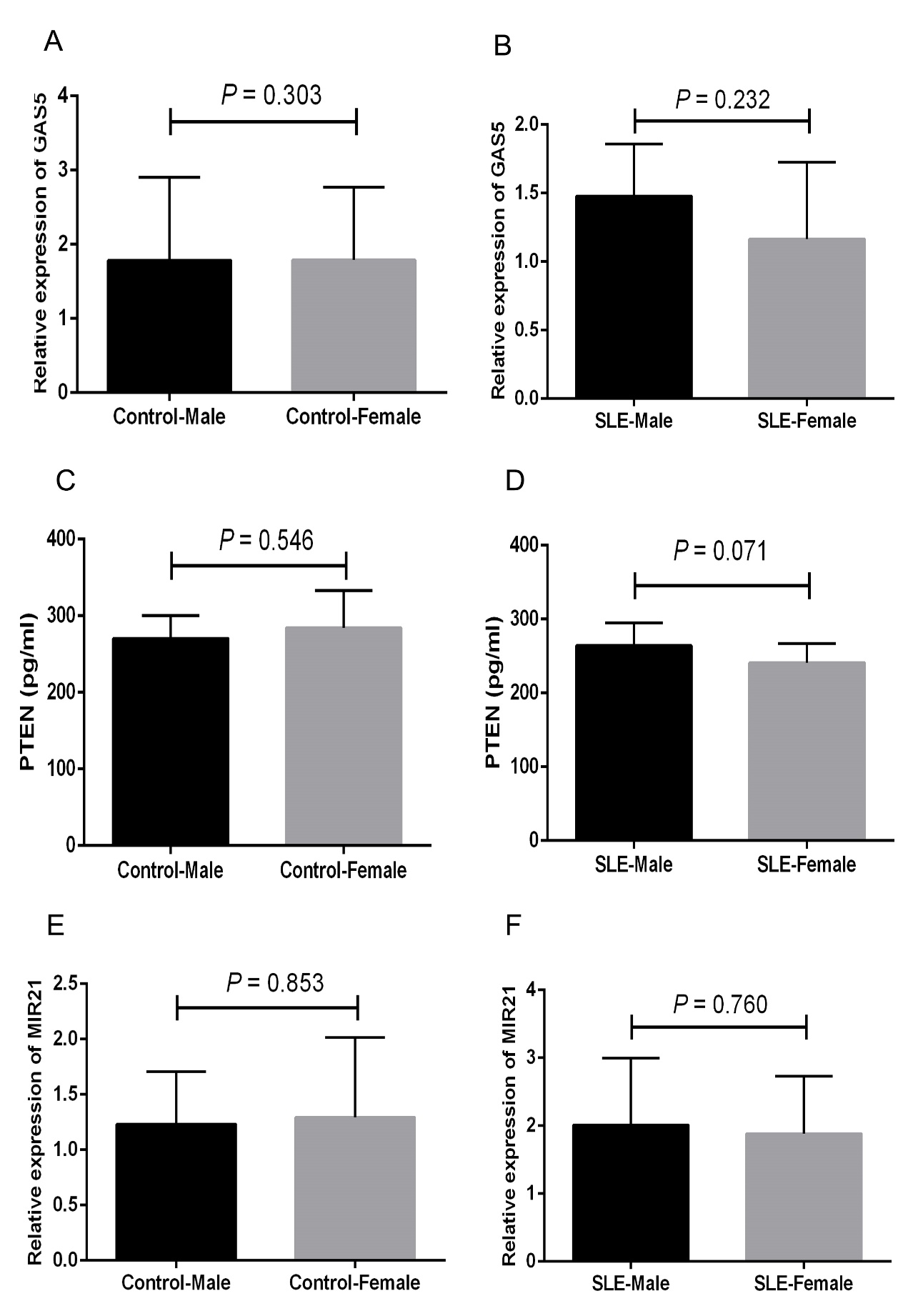


**Supplementary Figure 4.** The expression levels of GAS5, PTEN and miR-21 in the controls and SLE patients in male and female.
